# Analgesic Efficacy of Single-Shot Erector Spinae Block in Video-Assisted Thoracoscopic Surgery: A Propensity Score-Matched Retrospective Cohort Study

**DOI:** 10.7759/cureus.69795

**Published:** 2024-09-20

**Authors:** Wen Xuan Xu, Nancy Liu, Mella Y Kim, Xiaoyu Wu, Joobin Sattar, Kamal Kumar, Cheng Lin

**Affiliations:** 1 Schulich School of Medicine and Dentistry, Western University, London, CAN; 2 Anesthesiology and Perioperative Medicine, London Health Sciences Centre, London, CAN; 3 Anesthesiology, Western University, London, CAN

**Keywords:** acute pain management, erector spinae plane block (espb), minimally invasive surgical procedures, ultrasound-guided regional anesthesia, video-assisted thoracoscopic surgery (vats)

## Abstract

Introduction

Video-assisted thoracic surgery (VATS) is a minimally invasive surgical technique though effective analgesia remains a challenge. Erector spinae plane block (ESPB) has gained popularity due to its ease and safety of placement. In this study, we evaluated the analgesic efficacy of ESPB in patients undergoing VATS through a propensity score-matched retrospective cohort study. The primary outcome is the total opioid use in the first 12 postoperative hours.

Methods

We used binomial logistic regression to model whether patients received ESPB as a function of age, sex, body mass index (BMI), American Society of Anesthesiologists (ASA) physical status, and surgery type to generate a propensity score for each patient for matching.

Results

After screening 286 patients, 55 patients each in the ESPB and no-block groups were matched. ESPB was associated with a 1.2 mg (95% CI: -2.2 to -0.2) reduction in opioid use in IV hydromorphone equivalents when compared to no block. However, there was no reduction in the 12-hour pain score area under the curve or incidence of complications between the two groups.

Conclusions

ESPB was associated with a modest reduction in total opioid consumption although not a difference in pain score. While its analgesic efficacy may be limited, ESPB could be considered a component of multi-modal analgesia in VATS.

## Introduction

Over the last decade, minimally invasive video-assisted thoracic surgery (VATS) has gained prominence over thoracotomy [1]. Compared to thoracotomy, VATS is associated with a number of benefits: reduced postoperative pain [2-4], improved recovery [2,3], reduced morbidity [1-3], and more efficient use of hospital resources through shorter operating time [5] and shorter hospital stays [1-4].

Despite its “minimally-invasive” nature, patients undergoing VATS still experience moderate to severe pain [3,6] due to a multitude of sources, including injury to nerves, soft tissue injury, and irritation from chest drains. Furthermore, poor pain control can lead to pulmonary complications in this population who commonly have pre-existing impaired respiratory status [6]. Moreover, the risk of chronic postsurgical pain was reported as high as 25% in VATS [7,8], which can significantly impair quality of life. Therefore, ensuring adequate pain relief remains a priority even for VATS.

To control the multiple sources of pain associated with VATS, multimodal analgesia including regional anesthesia is routinely offered [9]. While thoracic epidural analgesia (TEA) [10-12] has traditionally been utilized for thoracic surgery, it has a number of disadvantages such as its invasiveness, high failure rate, and demanding technicality [9]. Additionally, TEA is associated with rare but significant complications, such as pneumothorax and spinal cord injuries [13,14]. Other reported complications, including hypotension, urinary retention, and lower limb weakness, may furthermore lead to delayed recovery and prolonged hospital stay [9].

Recently, interfascial plane blocks such as erector spinae plane block (ESPB) and serratus anterior plane block (SAPB) emerged as a popular analgesic modality for VATS due to their ease of placement [15-17], improved safety when compared to TEA [18], and potentially greater effectiveness than routine systemic analgesia alone [19]. The site of action of ESPB remains debated, but it is postulated to target both the dorsal and ventral rami of the thoracic spinal nerves, covering the deep muscles of the back as well. Therefore, ESPB is a promising regional anesthesia in VATS.

Although a few studies have evaluated the analgesic efficacy of ESPB in VATS, the results were inconclusive [20-23]. Therefore, our aim was to determine whether ESPB in patients undergoing VATS is associated with reduced opioid consumption in the first 12 postoperative hours through a retrospective propensity score-matched cohort study.

We presented this study as an abstract at the 2024 Canadian Anesthesiologists' Society Annual Meeting in June 2024.

## Materials and methods

This single-center retrospective study was approved by the Western University Health Science Research Ethics Board (ID: 112452). Data were extracted from the institution's electronic medical record and nursing charts by the co-investigators (Xu, Liu, Kim, Wu).

Population

This study includes patients who had undergone VATS procedures at Victoria Hospital, a tertiary academic center in London, Canada, from December 1, 2018, to January 1, 2020. All patients who had VATS with or without ESPB were reviewed for eligibility. Patients included were those aged 18 years or older, with American Society of Anesthesiologists (ASA) physical status I-IV, undergoing unilateral VATS, and receiving either an ESPB or no block. Exclusion criteria included BMI >45, chronic pain conditions, daily opioid use >60 mg of oral morphine equivalents, bilateral surgery, conversion to open thoracotomy, postoperative admission to intensive care unit, neuraxial nerve block or other type of block administration (i.e., not ESPB), and history of malignant hyperthermia.

Anesthesia

Patients in both groups received general anesthesia. Patients were induced with fentanyl 2-3 mcg/kg, lidocaine 1 mg/kg, propofol 1-2 mg/kg, and rocuronium 0.6 mg/kg, followed by intubation, generally with double lumen tube and rarely bronchial blocker. Patients were maintained on an air and oxygen mixture to maintain saturation of 92% and above on sevoflurane. Patients received intraoperative IV hydromorphone with the total amount at the discretion of attending anesthesiologists. We did not include intraoperative opioid use as this information was not readily available. Postoperatively, patients received hydromorphone PCA until chest tubes were removed, usually on postoperative day two.

For those in the ESPB group, patients received preoperative ESPB in the block room 30 minutes before surgical anesthesia. Following 1-2 mg of IV midazolam, ropivacaine 0.5% 20 mL was injected between the periosteum of the T4 transverse process and erector spinae muscle under ultrasound guidance using an in-plane technique. Aside from the ESPB, both groups received the same perioperative care.

Measured outcomes

The primary outcome was the total opioid consumption in IV hydromorphone equivalents, including self-administered PCA and nurse-administered parenteral and oral opioids, in the first 12 postoperative hours. The secondary outcomes included the area under curve (AUC) of the numeric rating scale for pain, assessed hourly in the first 12 postoperative hours, incidence of hypoxia during the first 12 postoperative hours defined by oxygen saturation below 90% with or without supplemental oxygen, duration of post-anesthetic recovery unit (PACU) stay, and the total length of hospital stay. The 12-hour period was chosen as a portion of patients were discharged before 24 hours, and many did not have pain scores and vitals collected between the 12 and 24 postoperative hour period.

Statistical methods

G*Power (version 3.1.9.2; Heinrich Heine University Düsseldorf, Germany) was utilized for power calculation. The sample size was calculated with α=0.05 and β=0.8, using a mean and standard deviation of opioid consumption in the first 12 postoperative hours in intravenous (IV) hydromorphone equivalent to 7.69±3.40 mg based on an internal audit of 20 patients. We considered a 30% change in opioid consumption to be a significant difference. Based on these parameters, 70 patients, 35 in each arm, were required. However, given the nature of a retrospective review, to reduce potential bias, all patients who had VATS with or without ESPB were reviewed for eligibility.

Statistical analysis was done using R (version 4.1.2; R Development Core Team, Vienna, Austria). Pre- and post-match patient demographics, including age, sex, BMI, ASA score, and type of surgery, are presented as median and standard deviation (SD) or numbers and percentages. Propensity score matching was used to deal with potential confounders. We used binomial logistic regression to model the anesthesia modality (general anesthesia vs. block + general anesthesia) as a function of age (<= 65 vs. > 65), sex (biological male vs. female), BMI (<= 40 vs. >40), ASA (2 and 3 vs. 4) and surgery type (wedge and segment resection vs. lobectomy and resection on multiple lobes) to generate a propensity score for each patient. Matching was done using the nearest neighbor method, 1:1 matching without replacement, and a caliper of 0.2 of logit. Unmatched patients were not included in the post-match analysis. The standardized difference in means was calculated for each covariate before and after matching to assess for balance. We considered a difference of <10% a balanced match. Continuous post-match variables were analyzed using Mann-Whitney U tests and categorical variables using Fisher’s exact tests.

## Results

**Figure 1:**
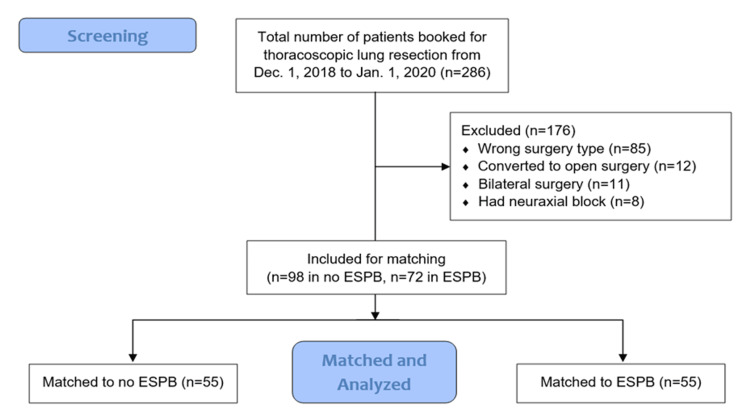
Flow chart for patient recruitment ESPB - Erector spinae plane block

Between December 1, 2018, and January 1, 2020, 286 patients were screened, with 170 ultimately meeting the criteria (98 in the no-block group and 72 in the ESPB group). Fifty-five patients in each group were successfully matched (Figure 1).

Patient demographics of each group before and after the match are noted in Table 1. Before the match, the standardized mean difference (SMD) showed some heterogeneity in age, sex, BMI, and surgery type. After the match, all covariates showed an SMD of less than 10%.

**Table 1:** Pre- and post-match baseline demographic characteristics SD - standard deviation; BMI - body mass index; ASA - American Society of Anesthesiologists, SMD standardized mean difference

	Pre-match			Post-match		
	No Block	ESPB	SMD (%)	No Block	ESPB	SMD (%)
Sample size, n	98	72		55	55	
Age (years)						
Mean ± SD	66.2 ± 11.9	64.2 ± 12.3		67.6 ± 11.4	65.0 ± 13.3	
> 65.0 (n, %)	62, 63.3%	39, 54.2%	18.3	34, 61.8%	34, 61.8%	0.0
BMI (kg/m^2^)						
Mean ± SD	28.9 ± 7.2	28.6 ± 6.9		28.7 ± 6.5	28.0 ± 6.3	
> 40 (n, %)	5, 5.1%	4, 5.6%	19.8	1, 1.8%	1, 1.8%	0.0
Sex (n, %)			16.9			7.3
Male	60, 61.2%	38, 52.8%		33, 60.0%	31, 56.4%	
Female	38, 38.8%	34, 47.2%		22, 40.0%	24, 43.6%	
ASA (n, %)			9.4			3.9
2	5, 5.1%	1, 1.4%		0, 0.0%	0, 0.0%	
3	66, 67.3%	48, 66.7%		41, 74.5%	42, 76.4%	
4	27, 27.6%	23, 31.9%		14, 25.5%	13, 23.6%	
Surgery type (n, %)			40			4.0
Wedge	41, 41.8%	20, 27.8%		17, 30.9%	19, 34.5%	
Segmentectomy	7, 7.1%	2, 2.8%		3, 5.5%	2, 3.6%	
Lobectomy	33, 33.7%	30, 41.7%		20, 36.4%	24, 43.6%	
Surgery on Multiple Lobes	17, 17.3%	20, 27.8%		15, 27.3%	10, 18.2%	

ESPB was correlated with a reduction of 1.2 mg (95% confidence interval (CI): -2.2 to -0.2 mg, p=0.02) in 12-hour opioid use in IV hydromorphone equivalents. E-value, which measures the robustness of the result against unaccounted confounders, for the primary outcome was 1.7 (lower 95% CI: 1.2). There was no associated difference in 12-hour pain score AUC, PACU length of stay, hospital length of stay, or incidence of hypoxia between the two groups (Table 2).

**Table 2:** Measured outcomes in pre- and post-matched samples with differences in mean and odds ratios AUC - under the curve (AUC) ^a^Median and interquartile range ^b^N and % ^c^Result computed with Mann-Whitney U test ^d^Result computed with Fisher’s exact test

	No Block	ESPB	Difference and 95% CI^c^	P value
12 Hour Opioid Use^a ^(mg)	4.0 [2.2-6.2]	2.8 [1.5-4.1]	- 1.2 mg [-2.2 to -0.2]	0.020
12 Hour Pain AUC^a^	4.0 [2.0-5.5]	3.8 [2.5-5.0]	- 0.2 [-1.1 to 0.6]	0.60
PACU Length of stay (min)	160.0 [118.0-180.0]	156.0 [113.5-203.5]	- 4.0 [-20.0 to 27.0]	0.75
Hospital Length of stay (day)	3.0 [2.0-4.0]	2.0 [1.5-3.0]	0.0 [-0.0 to 0.1]	0.25
			Odds Ratio and 95% CI^d^	P value
Hypoxia^b^	6, 10.9%	3, 5.5%	0.47 (0.07 to 2.37)	0.50

## Discussion

Poorly controlled acute pain post VATS can result in chronic postsurgical pain in as high as 25% of patients [7,8]. Therefore, adequate early analgesia is essential in VATS. While our study showed an associated reduction in the first 12-hour opioid consumption in the ESPB group, this reduction was modest and clinically insignificant. This was not unexpected. While the ESPB may target pain mediated by thoracic spinal nerves, irritation of the diaphragm, visceral pleura, and more distant thoracic dermatomes may not be covered by ESPB.

Other studies have demonstrated a more pronounced reduction in postoperative analgesia use within the first 24 hours when compared against with no block group [19,24] or a placebo saline block group [25]. Cifti et. al conducted a prospective randomized study that showed that single-shot ESPB using bupivacaine was associated with consistently lower opioid consumption in the first 24 postoperative hours compared to no block [19]. Two similar randomized control trials using ESPB single-shot ropivacaine also showed reduced sufentanil consumption, both intra- and post-operatively [24,25].

Although we did not observe a difference in the first 12-hour AUC in pain score in our study, Cifti et. al showed that single-shot of bupivacaine was associated with lower passive and active pain scores compared to no block, for the first 24 postoperative hours [19]. Multiple other studies also showed a significant reduction in pain scores with ESPB in comparison to no block, sham, or deep SAPB for at least the first six to eight postoperative hours [24,26-28]. This difference in findings could be due to multiple factors, including variations in ESPB administration (dosing, level, and timing) or pain score measurement.

Moreover, effective analgesia in VATS can promote recovery through early mobilization and reduce postoperative complications [3]. Cifti et. al demonstrated that the use of ESPB is associated with faster postoperative out-of-bed activity compared to no block [19]. ESPB has also been associated with earlier discharge times when compared with sham block [25,27]. Furthermore, patients reported higher satisfaction with ESPB compared to placebo saline blocks [25]. Based on these findings, ESPB appears to improve postoperative recovery, and future studies examining postoperative outcomes in patients undergoing VATS would be warranted.

On the other hand, despite the fact that we and others have shown ESPB to be more effective than no block, the best regional anesthesia technique remains debated. ESPB has been shown to be less effective than thoracic paravertebral blocks and no more effective than intercostal nerve blocks for postoperative morphine consumption [11,20]. A network meta-analysis by Sandeep et. al in 2022 showed that thoracic paravertebral blocks are superior to ESPB in reducing IV morphine use postoperatively [28]. Therefore, further studies comparing ESPB against other regional blocks are needed to reach a consensus.

As a retrospective study, there may be unaccounted confounders. The E-value of 1.7 suggested the data may be weak against unmeasured biases. Self-reported pain scores are also subjective and can vary due to differences in pain tolerance between patients, which may be a confounding variable when evaluating the effectiveness of ESPB. A more standardized alternative could be the QoR-15 score, which is a multilingual postoperative quality of recovery measurement tool validated through a meta-analysis [29]. One important confounder we were not able to balance was intraoperative opioid use. This may contribute to the weak E-value. Due to a significant proportion of patients being discharged between 16 and 24 hours postoperatively, outcomes in and beyond this interval were thus not collected but would otherwise provide important clinical information. In addition, there may have been changes over time in anesthesia and surgical technique that we did not account for. Our study was only conducted using participants from one hospital, which may limit the generalizability of our findings. Since ESPB is a relatively new type of block, further studies should be conducted at centers participating in the use of this technique. Finally, given the time limitations of our data collection, we did not assess functional outcomes such as time until ambulation or chronic pain outcomes.

## Conclusions

ESPB is an attractive component of multimodal analgesia for VATS due to its ease of placement and low incidence of complication. Our retrospective propensity score-matched cohort study showed that ESPB was associated with a very modest reduction in postoperative opioid consumption. No difference was detected in the first 12-hour pain score AUC, incidence of postoperative hypoxia, length of PACU stay, or length of hospital stay between the ESPB group and the non-block group. Given a number of emerging paraspinal blocks such as retrolaminar blocks and intertransverse process blocks are gaining popularity, future studies can be directed to compare the analgesic efficacy of such blocks.
